# A framework for understanding how activities associated with dog ownership relate to human well-being

**DOI:** 10.1038/s41598-020-68446-9

**Published:** 2020-07-09

**Authors:** Ana Maria Barcelos, Niko Kargas, John Maltby, Sophie Hall, Daniel S. Mills

**Affiliations:** 1 School of Life Sciences, University of Lincoln, Lincoln, UK; 2 School of Psychology, University of Lincoln, Lincoln, UK; 3 Department of Neuroscience, Psychology and Behaviour, University of Leicester, Leicester, UK

**Keywords:** Psychology, Emotion, Social behaviour, Stress and resilience, Quality of life, Psychiatric disorders

## Abstract

There is notorious inconsistency regarding mental health benefits of dog ownership, partially due to repeated cross-sectional studies comparing dog owners and non-owners, without taking into account the heterogeneity of dog-owner dyads, especially the activities with which the owners are involved. This study aimed to develop a comprehensive framework of the most important dog human related activities and their impact on owner well-being. Six focus groups with 35 dog owners were conducted, and their audio transcripts thematically analysed. Dog human related activities and themes of activities were linked to their reported changes in well-being through matrix coding. A framework of 58 dog human related activities linked with their specific hedonic well-being, life satisfaction and eudaimonic well-being outcomes was generated. Most activities were reported to improve owner’s well-being, (e.g. human–dog tactile interaction increases owner’s self-esteem), and a minority was mainly associated with negative outcomes. The richness of the framework presented in this study reinforces the importance of assessing dog ownership well-being outcomes based on specific dog human related activities with which dog owners are involved. This new and systematic investigative approach should decrease inconsistencies in the field and facilitate mental health interventions and study designs of a higher level of evidence.

## Introduction

Mental health problems are one of the main disease burdens of society and are growing worldwide^1^. In the United Kingdom, mental health problems represent the largest single cause of disability, with estimated costs of £105 billion a year; one in four adults in the country suffers at least one diagnosable mental health problem in any given year^2^. Psychological changes led by pet ownership may have an important impact on mental health, with associated economic savings (£2.5 billion/year—UK)^3^. However, studies in this field are inconsistent, and how pet ownership might impact on human well-being has not been explored systematically. Heterogeneity within important aspects of pet ownership (e.g. amount of exercise undertaken, level of disclosure of personal emotional information with their dogs) may explain why some individuals may benefit while others do not^4–6^. It is therefore not surprising that investigations on depression have shown pet ownership improves^7–9^, as well as makes no difference^10–12^ and even worsens the condition^13,14^. Similar contradictions extend to other aspects of well-being, such as loneliness^8,15^, stress^13,16^, anxiety^13,17^, human functioning^11,18^ and life satisfaction^19,20^.

The tendency to compare ‘pet owners’ versus ‘non-owners’ in cross-sectional research^21^ is a gross oversimplification of a complex relationship. Clearly, the specific activities owners engage with, rather than the simple act of ownership is important. 35% of dog owners, for example, do not walk their dogs^22,23^, and so benefits associated with increased exercise cannot be expected in this subpopulation; likewise, variations in time spent with the pet are likely to change life satisfaction and anxiety in owners^17^. Where dog-ownership related activities have been considered, the research approach has tended to be top-down (i.e. dictated by the preconceived ideas of the researcher), focusing on the impact of a few specific activities. Dog walking, for example, has been extensively investigated and linked with several changes in well-being, such as increase in social interactions^15,24–27^, social support^24,26–28^, human functioning^29,30^, feelings of happiness^31^, relaxation, anxiety, stress^24^, annoyance^26^. However, it should be recognised that owners may attribute a wider range of beneficial outcomes to these activities, and it is only by questioning them that the breadth of potentially important activities is likely to be identified (i.e. using a bottom-up approach).

**Figure 1:**
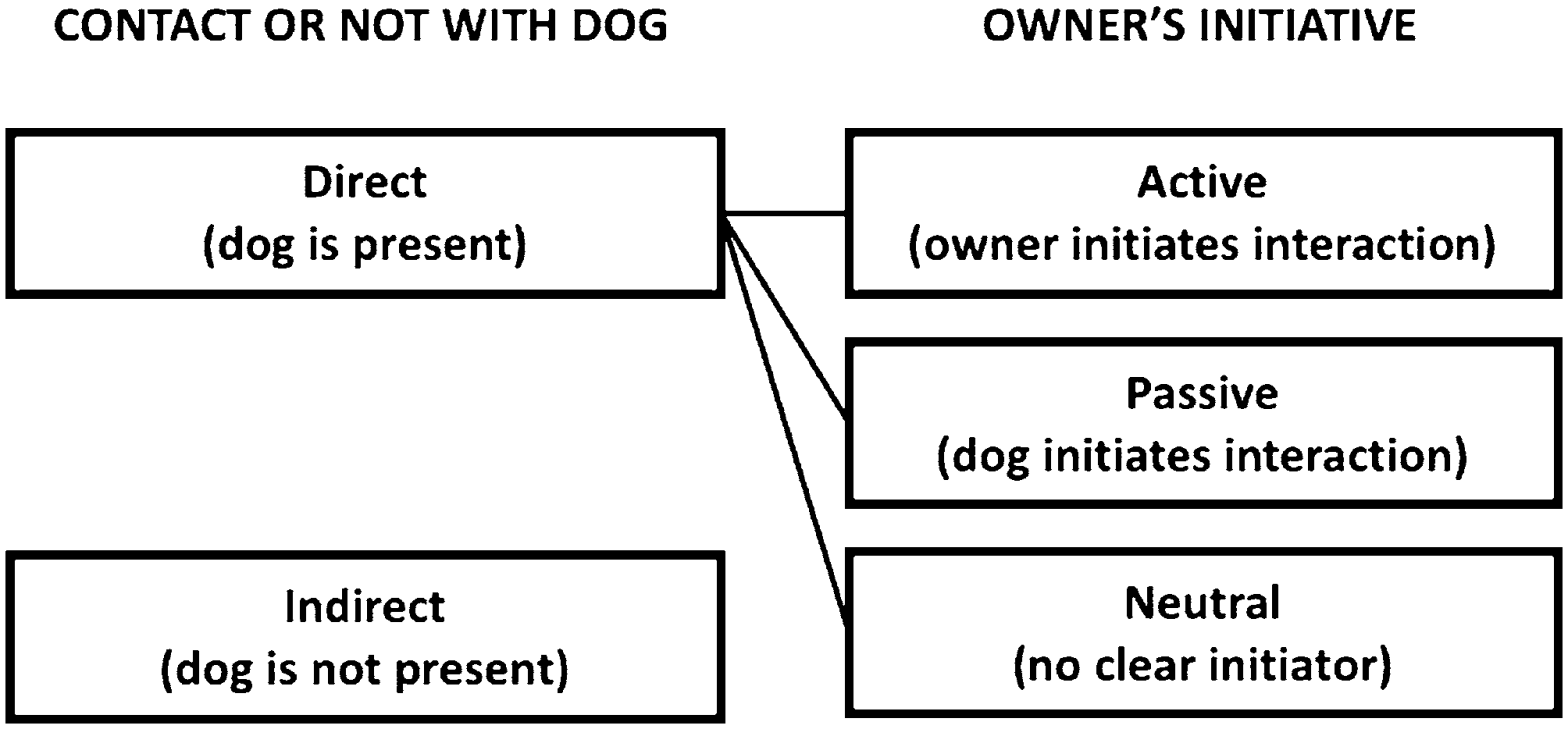
Dog human related activities classification scheme.

There appears to be a lack of systematic consideration of *the full spectrum of specific activities/events that occur due to the dog’s existence in the person’s life which may be of relevance*. We define these activities as *dog human related activities (DHRA)*, also referred to simply as ’activities’ within the article (Fig. 1). This definition guided the design of this study and may direct future studies in dog–human interaction. Here, we propose classifying activities as either direct (occurring in the presence of the dog), such as walking the dog, petting it, the mere company of the dog; or indirect (do not require the presence of the animal), e.g. buying food for the dog, meeting dog friends, studying dog-related topics. Direct activities are not restricted to events initiated by the owner (active activities, e.g. grooming, feeding), but also include interactions started by the pet (passive activities, e.g. jumping up, licking the owner, barking) and neutral events, in which it is not clear who initiates the interaction (neutral activities, e.g. the company/presence of the dog, sleeping next to each other, watching tv together). Only with a comprehensive list of these activities, can we hope to identify the important relationships that might improve human well-being.

Well-being is frequently examined using a hedonic and eudaimonic perspective. Hedonia is a focus on the experience of pleasant events, while eudaimonia focuses on a fulfilling life aimed at achieving one’s potential^32^. Hedonic well-being is composed of positive and negative affect but is frequently operationalised as subjective well-being (SWB), a broader term comprising affect and life satisfaction^33^. Affect (or core affect) is the manifestation of moods and emotions^34^, and life satisfaction is the judgment of one’s own life, e.g. feeling delighted or terrible about life^35^. In contrast, eudaimonic well-being, also referred to as psychological well-being (PWB), is typically composed of six elements or areas of functioning: (1) autonomy, one’s independence and freedom from others’ approval, (2) environmental mastery, one’s fit and mastery on surrounding environment, (3) personal growth, one’s self-realization and achievement of one’s potential, (4) positive relations with others, one’s feelings of empathy and affection for others and good social relations, (5) purpose in life, one’s meaning in life and comprehension of one’s purpose, (6) self-acceptance, one’s positive self-regard with acceptance of past life and good and bad qualities^36^.

In this study, we used dog owner-generated data from focus group sessions to systematically identify the most important dog human related activities (both direct and indirect activities) impacting on self-perceived human well-being (hedonia, life satisfaction and eudaimonia) in order to develop a comprehensive framework, which lays the groundwork for future quantitative studies.

## Results

A framework of dog human related activities and their reported well-being outcomes in hedonia, life satisfaction and eudaimonia has been generated from the focus group sessions with an heterogenous sample of dog owners representing owners of different ages, genders, dog-related expertise, having dogs of various sizes, ages and with varying durations of dog ownership. A general overview of the framework is provided in the section below, while more details of the framework are provided in the subsequent sections in the following order: first, activities related to the four main aspects of hedonic well-being, second, activities related to life satisfaction and, third, activities related to the six elements of eudaimonic well-being. Percentages provided within the Results section are purely a description of the data generated and should not be used for quantitative analysis.

### Dog human related activities and well-being outcomes

**Figure 2:**
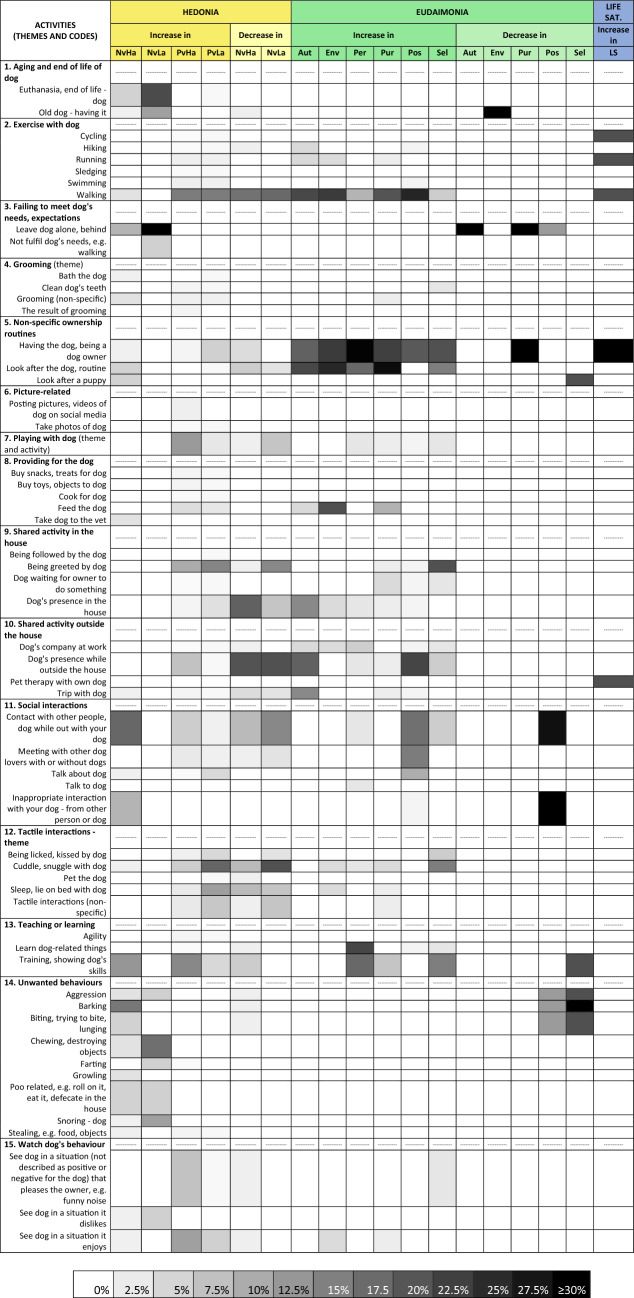
Heat map of all dog human related activities and their respective well-being outcomes. The darker the colour in the cell higher is the relative frequency of mentions of the activity (rows) in relation to the well-being outcome in the column. E.g. ‘looking after the dog’ (dark cell) was mentioned to increase purpose in life more times than ‘running with the dog’ (light cell). **Nv** and **Pv** (negative and positive valence, respectively), **Ha** and **La** (high and low arousal, respectively), **Aut** (autonomy), **Env** (environmental mastery), **Per** (personal growth), **Pur** (purpose in life), **Pos** (positive relations), **Sel** (self-acceptance), **LS** (life satisfaction).

A total of fifty-eight activities were reported as important for participants’ well-being. They were divided into 15 themes (Fig. 2). The most commonly mentioned themes were: ‘exercise with dog’ (count: 127, 14.9%), especially walking; ‘non-specific ownership, routines’ (120, 14.1%), mainly having a dog and looking after it; ‘tactile interactions’ (100, 11.7%), notably cuddling/snuggling with dog; ‘social interactions’ (91, 10.7%), such as the contact with other people/dog while out with dog; ‘shared activity in the house’ (75, 8.8%), mainly being greeted by dog; ‘shared activity outside the house’ (75, 8.8%), especially the presence/company of the dog; and ‘teaching or learning’ (66, 7.7%), mainly training the dog.

Eighteen well-being outcome themes also emerged for the framework (Fig. 2). Aspects of hedonia most frequently associated with dog human related activities were an increase in ‘positive valence-high arousal’ states (195 mentions, 22.9%), such as excitement, happiness, and an increase in ‘positive-valence-low arousal’ states (136, 16.0%), e.g. calmness, relaxation. For eudaimonia, ‘positive relations with others’ (93, 10.9%), such as social interactions, and ‘purpose in life’ (57, 6.7%) prevailed.

Details of all well-being outcomes and the dog human related activities reported to lead to them are given in the heat map in Fig. 2. The darker the cell in the map, the higher the percentage of mentions (up to ≥ 30%) of the relevant activity within the well-being outcome column.

### ‘Negative valence-high arousal’ and dog human related activities

In this aspect of hedonic well-being negative feelings such as ‘annoyed’, ‘angry’, ‘stressed’, ‘worried’, and ‘frustrated’ emerged. Increase in feelings of this aspect were mainly occasioned by unwanted dog behaviours, such as barking; but also more benign social interactions with dog/people, especially negative encounters while out with their own dog. For example, participant 15:

It is quite annoying when people don’t ask if a dog is friendly first, you shouldn’t just go straight and stroke the dog, you shouldn’t be touching a dog if he has an owner.

Also, teaching or learning dog-related things, particularly training the dog; and non-specific ownership routines, such as looking after a puppy, had a negative impact on this aspect of well-being. Some activities, however, were reported as protective against these negative feelings, especially shared activities with the dog outside and inside the house, simply due to the dog’s presence/company in these contexts. Tactile interactions with the dog, such as cuddling and sleeping together, and exercises with the dog, notably walking, also played a key role in decreasing feelings of negative valence-high arousal. For example, participant 4:

It is very hot in [name of the country] so I jog at night, like at midnight, so I take her with me, it makes me feel protected, it is quite nice. She gives me company, like someone is with me, so I don’t have to be afraid, it reduces my fear.

### ‘Negative valence-low arousal’ and dog human related activities

This aspect of hedonic well-being included states such as ‘sad’, ‘tired’, ‘unhappy’, ‘lonely’, ‘depressed’. Elements of this aspect increased in dog owners mostly when they felt they failed to meet their dog’s expectations/needs, especially for leaving the dog alone/behind, and when their dogs performed an unwanted behaviour, notably the destruction of objects. For example, participant 25 said:

If it [destruction of object] is in the middle of the day it is very tiring, it is not even anger, you are exhausted.

Additionally, having an old dog and coping with the end of life of the animal, such as euthanasia, was associated with negative feelings within this aspect. Negative emotions of low arousal were nevertheless improved by tactile interactions with the dog, such as cuddling, and by sharing activities with the dog both inside the house, especially by being greeted by the animal, and outside the house, in particular, the dog’s presence/company. Also, exercising together, mainly walking, was frequently reported to decrease these negative feelings. For example participant 16, explained:

It was difficult at times to walk him, but I was determined, me and him. For me, walking is on the higher top of my list, it helped in my recovery [from depression]

### ‘Positive valence-high arousal’ and dog human related activities

This includes states such as ‘happiness’, ‘joy’, ‘fun’, ‘excitement’, ‘activation’. Owners reported improvements in feelings of this aspect from exercising with their dogs, especially walking; from watching their dog, mainly when the dog is happy in a situation; and from teaching the dog, such as training exercises. For example, participant 7:

It makes me happy to see how happy she [the dog] is, how excited she is.

Positive feelings of high arousal also arose from tactile interactions with the dog, such as cuddling and lying next to each other, playing with the dog, e.g. tug and ball games; and shared activities inside the house, notably being greeted by the dog. No activity was identified to directly decrease this element of well-being.

### ‘Positive valence-low arousal’ and dog human related activities

In this aspect of well-being, feelings of calmness, relaxation, peace, and love emerged. They reported growth in feelings of ‘positive valence-low arousal’ when they were involved in tactile interactions with their dogs, such as cuddling, sleeping together and lying next to each other. For example, participant 1:

Snuggling with the dog, on the sofa or on the bed, they just come up and flop on you, you can just relax and forget how busy it is and things like that, it is really calming.

Additionally, exercising with the dog, mainly walking, and sharing activities in the house, notably being greeted by the dog, were also associated with increases in feelings of this aspect. No activity was reported to directly decrease this element of well-being.

**Figure 3:**
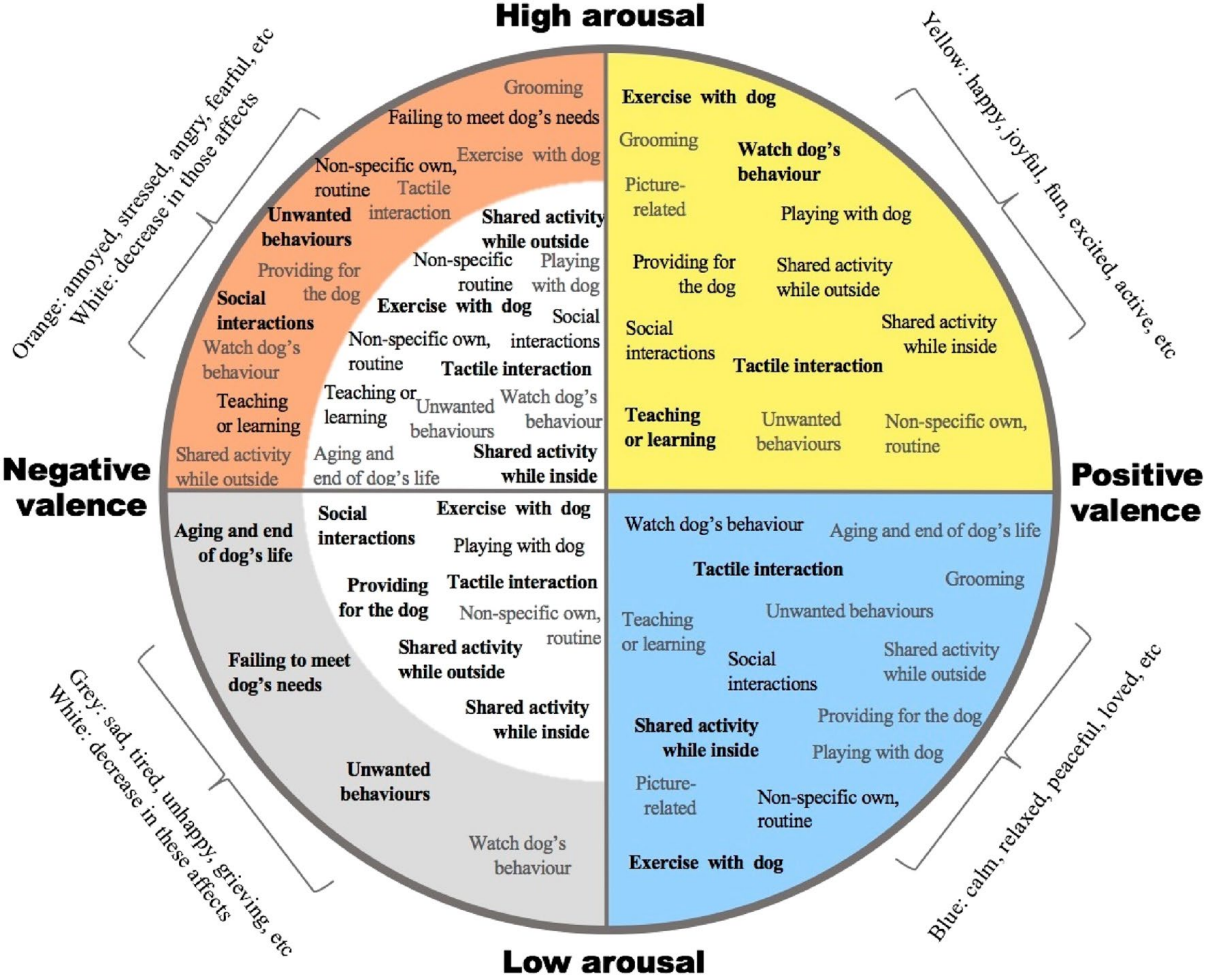
Model of impact on affect (valence and arousal) for all themes of dog human related activities. Each coloured quadrant contains themes of activities that increase an aspect of affect, while white areas contain themes that were reported to cause reduction of that aspect of affect. The spatial position of items within quadrants is not related to any difference in intensity. Themes in **bold**: had a high frequency of mentions in the well-being element reported (10% or more). Black: moderate frequency of the mentions (5.0–9.9%). Grey: low frequency of the mentions (0.01–4.9%).

The results relating to dog human related activities effects on the valence-arousal (hedonic well-being) are summarised in Fig. 3.

### ‘Life satisfaction’ and dog human related activities

There were not many mentions of life satisfaction as an outcome of dog human related activities. The few mentions that did occur tended to be related to exercising with the dog, such as walking, running and cycling, dog ownership per se, general routine with the dog and taking the animal to animal-assisted interventions. For example, participant 16:

He [the dog] was life changing, he improved my life and the life of my family, he has a positive impact on the whole family.

### ‘Autonomy’ and dog human related activities

In this element of eudaimonic well-being, ‘non-specific ownership routines’, such as being a dog owner and being able to look after an animal were frequently mentioned by owners as activities that make them feel more autonomous. For example, participant 8:

As a pet parent I do feel protective of him and I have that sense that I can go out and take care of him, so I feel independent.

Increases in this element were also associated with shared activities with the dog (outside and inside the house), especially having the dog’s presence/company; and with exercises with the dog, such as walking, running, hiking. In contrast, owners’ autonomy decreased when they struggled to leave their dog behind/alone. For example, participant 5:

Sometimes dogs restrict that [autonomy, independence], you don’t always have the opportunity to take dogs somewhere, we have a family in [another country far away] and to go and travel there means you have to leave the dog somewhere, and sometimes there is no place to leave the dog.

### ‘Environmental mastery’ and dog human related activities

In this element, owners reported their ability to fit or cope with their surrounding environment, including the performance of tasks relating to daily living. Improvements in environmental mastery were associated with dog ownership and looking after the animal on a regular basis, which is part of the theme ‘non-specific ownership routines’.

Also, performing exercises with the dog, mainly walking, and providing for the animal, particularly feeding it, were reported to increase the sense of environmental mastery of owners. For example, participant 10:

It [feeding the dog] gives me something to do, otherwise I would skip a few meals. They have their own dinner, they have their own routines so I need to keep my routines as well. They have routine in the morning, it is good, it reminds me of doing things. One of the dogs has tablets, so it reminds me of my tablets as well.

However, owners also mentioned that having an old dog and giving support to this old animal hindered their own environmental mastery. For example, participant 31:

The negative side of dog ownership is when they get old, you need to adjust your life to help your dog, […] you need to adjust loads of your routine.

### ‘Personal growth’ and dog human related activities

Personal growth was reported as a sense of self-achievement in particular aspects of the owner’s life (or life as a whole). The activities that mainly contributed to this element of well-being were being a dog owner and being capable of looking after a dog. Also, teaching and learning were associated with personal growth, especially learning dog-related things, such as dog behaviour and dog travel regulations, and training the dog successfully, e.g. to perform a new skill. For example, participant 35:

He is the first dog I have had as an adult so I had to learn something about dog behaviour, how to look after them, about visas, how to get a [nationality of dog] dog into this country, that helped my personal growth.

No activity was reported to decrease this element of well-being.

### ‘Purpose in life’ and dog human related activities

Having a dog, looking after the animal and having a routine because of the dogs’ needs were associated with improvements in their purpose in life. Additionally, exercising with the dog, primarily through walks, increased this element as well, as did some non-specific activities; for example participant 23:

Because I got a dog I got quite a good routine now. I need to make sure she walks, she is fed, all the needs are met, so that gives me purpose in life.

Some activities, however, decreased or hindered owners’ sense of purpose in life, such as the responsibilities linked to dog ownership and not being able to leave the dog behind/alone. For example, participant 33:

If I didn’t have my dogs I would travel more and be more free, I would do more things, it is more negative to purpose in life, not autonomy, because I think in a big picture.

### ‘Positive relation with others’ and dog human related activities

Dog owners reported changes in their social relations and empathy for others. Increases were attributed to having more interactions with other people, especially when out with the dog, and explicitly meeting other dog lovers on purpose. Exercising with the dog, particularly walking, and being accompanied by the dog while outside the house improved owners’ relations with others as well. For example, participant 20:

When you go out for a walk you meet different people, it seems okay to talk to them because you have a dog, the dog is an introducer. If you are just walking by yourself it is different, the dog is the connector.

Several owners also said that simply having a dog was beneficial to their social relations. In contrast, contact with others was also detrimental to their social relations, especially when having disruptive encounters, such as inappropriate interactions from others towards their dogs. Unwanted behaviours, particularly those related to aggression, such as when their dogs bark, bite or lunge towards others, were also detrimental to their social relations. For example, participant 25:

Sometimes it is annoying meeting other people. Yesterday, for example, there was a guy sitting with three dogs, and then a woman approached and tried to pet them, then all dogs started barking, and the guy was trying to calm the three dogs. Sometimes I think ‘please don’t interact with my dogs, just leave me’

### ‘Self-acceptance’ and dog human related activities

Self-acceptance included owners’ self-esteem and acceptance of their good and bad qualities. This element improved through the simple fact of having a dog and being able to look after the animal successfully, as well as due to activities shared with the dog in the house, particularly being greeted by the dog. For example, participant 2:

Being licked, being cuddled, greeted [by dog] helps with self-esteem and self-acceptance because you can feel that you are loved, like when you feel sad, they pick up that and they try to cheer you up, it helps you accept who you are.

Tactile interactions with the dog, such as cuddling and being licked/kissed by the dog, and teaching or learning dog related things, such as dog training and learning about dog behaviour, had a beneficial impact on this element as well.

By contrast, owners’ self-acceptance was hindered by dog’s unwanted behaviours, especially due to the feeling of not being able to control their animal’s behaviour, such as when the dog was acting aggressively or getting a recall when it was needed. For example, participant 27:

On training she is great but on public she won’t come back to me when I call her, then suddenly she starts to bark to someone with no reason, and I can’t control her, it makes me feel completely useless.

Training the dog and looking after a puppy also decreased owner’s self-esteem, particularly due to the frustration they felt for not being able to train a specific skill or manage their puppy.

**Figure 4:**
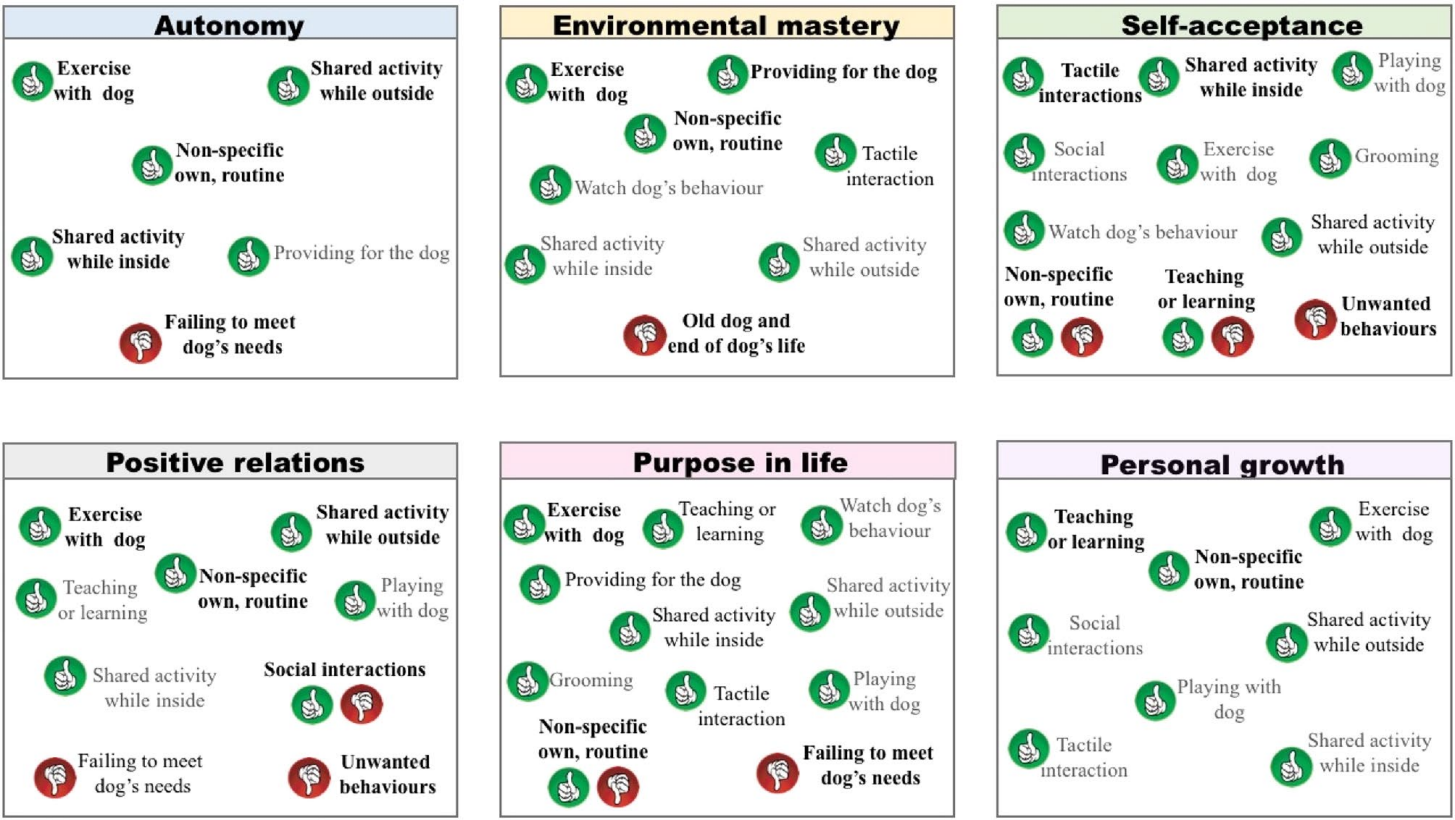
All themes of dog human related activities reported to impact on eudaimonic well-being. Themes in **bold**: high frequency of the mentions in the element of well-being reported (10% or more). Black: moderate frequency of the mentions (5.0–9.9%). Grey: low frequency of the mentions (0.01–4.9%). A green ‘thumbs-up’ indicates that the activity is beneficial to the referred well-being, while a red ‘thumbs-down’ shows the activity hinders that well-being. A few activities can be both beneficial and detrimental depending on the specific context of the interaction.

These results relating to the themes of dog human related activities associated with eudaimonic well-being are summarised in Fig. 4.

## Discussion

The recurrent inconsistencies observed across studies investigating dog ownership and human well-being, without taking into consideration the individuality of each dog–human relationship, drove the use of a new investigative approach in this study. A framework based on the full spectrum of activities/events dog owners could be involved with, due to the existence of a dog in their lives (defined by us as dog human related activities – see Introduction) was created. The use of the activities (or themes of activities) of our framework in future investigative studies is likely to increase consistency across studies, as patterns of well-being outcomes are expected to be observed for each particular identified activity. We believe this is the first systematic attempt to identify the most important specific dog human related activities that have an impact on the perceived well-being of owners. The framework is comprised of 58 dog human related activities and 15 themes of activities, which emerged as the most important dog human related events to dog owners’ hedonic well-being, life satisfaction and eudaimonic well-being. Importantly we considered not only positive but also negative impacts, since the balance between these may be critical in the final well-being outcome.

As might be expected, the majority of dog human related activities were associated with positive well-being outcomes but three important groups of activities had a predominantly negative impact on owners’ well-being: (1) aging and end of dog’s life, such as euthanasia, (2) failure to meet dog’s needs/expectations, such as leaving the dog alone at home, and (3) unwanted behaviours, such as problematic tendencies and poor obedience. The richness of the framework revealed in this study reinforces the problem with oversimplified dichotic comparisons based purely on ownership or not frequently observed in studies of the “pet effect”^21,37^. Our data support the need for better mapping of activity to outcome: for example, being greeted by a dog, physical interactions with the animal and dog training, were repeatedly referred to as boosters for owner’s self-acceptance, by contrast walking the dog had a stronger impact on owner’s social interactions, autonomy and purpose in life. It should also be noted that having to leave the dog alone at home, which may be related to owner work status, or looking after an old dog was related to important negative feelings of low arousal, such as sadness, and to decrease in autonomy or environmental mastery. In contrast, the occurrence of unwanted behaviours and obedience issues were predominantly linked with negative feelings of high arousal, such as anger, and worsening on positive relation with others and self-acceptance.

It is not surprising that the most frequently reported activities to impact on well-being in this study, have also been reported to be important in previous investigations. Physical interactions with dogs, for example, have been shown to decrease stress^38–41^, anxiety^42–44^ and fear^45^, and increase feelings of positive valence^15^. Being greeted by the dog is suggested to provide comfort and positive feelings for owners^46^. Dog walking often catalyses owners’ social interactions^15,47^, improves owner’s emotional state^24^ and gives them purpose in life^15^. In contrast, unwanted behaviours may hinder owners’ social interactions^46^ and generate feelings of negative valence^48^. The loss of a pet^46^ and the care of an old animal^49^ have also been linked to negative feelings of low arousal, such as sadness and depression, as has a sense of failure to fulfil the dog’s expectations, e.g. when owners fail to walk them^26^ or leave them alone at home. However, our study extends this list and systematically integrates this more comprehensive inventory of activities into a coherent framework for the first time (Figs. 2, 3 and 4).

Changes in affect (hedonia) were mainly reported to be caused by specific events, such as playing, training, being greeted by the dog, while changes in eudaimonic well-being (eudaimonia) were mainly linked to more general events, such as being a dog owner and looking after the dog. This difference is not surprising since hedonia (represented by affect) amounts to the pursuit of pleasure through the experience of discrete life events, responsible for changes in feelings^32,50^; by contrast, eudaimonia, represents a lifestyle, (“eudaimonic living”)^51^, in which intrinsic values and long-enduring outputs are aimed for^36^, making eudaimonia more stable over time than hedonic well-being^33,52^. Thus, it seems likely that either a set of activities or a high intensity/frequency/duration of activities is required to yield transformations in eudaimonia, rather than the experience of a single event. For example, walking a dog once a year may suffice to increase one’s momentary happiness and be identified as an activity that improves this affect. However, this same event may not be enough to change the meaning or quality of one’s life (aspects of eudaimonia).

Changes in life satisfaction, one of the components of subjective well-being, was not linked to dog human related activities by the majority of participants. This might be a product of the study design, which required participants to focus on the four most important dog human related activities for their affect and/or life satisfaction in the same exercise. This might have encouraged them to focus more on specific events and thus subjective feelings. Another possible limitation is the lack of males in the sample, even though an effort was made to increase their representation in the selection phase. Women are believed to have higher psychological openness than men^53,54^, thus, future studies may need to minimise the disclosure of information about the nature of the research to recruit more men. Nonetheless, the activities and changes in well-being reported by male participants in the focus group sessions were not noticeably different to those reported by their female counterparts, and sessions were continued until redundancy of the information provided by all participants was achieved, but this does not negate the possibility of a selection bias limiting the information provided.

To increase the consistency across future studies, we recommend the selection of individual activities provided in our framework or combinations of them to test associations with the potential well-being outcomes as identified here. For example, testing if a higher frequency (or duration) of tactile interactions with dogs is positively associated with self-esteem. However, if our list of activities does not mirror a specific targeted population (e.g. dog owners with physical disability; dog owners who live on farms), new specific activities (or themes) could be added to the existing framework to make it even more comprehensive or a new framework specific for this group could be created based on the specific dog human related activities of interest. By assessing individual activities, rather than dog ownership per se (or even dog-assisted intervention per se), future investigations are likely to identify patterns of increase or decrease in particular well-being outcomes in relation to specific activities, especially if representative sampling of their targeted population and control for potential mediators (e.g. demographics, closeness to pet) are applied. Thus, it might be evidenced that dog ownership per se is not the key to change elements of well-being but dog-related activities.

Future studies by the authors will quantitatively assess the relationship between dog human related activities and changes in well-being identified here, through a survey, as a prelude to more comprehensive investigations of causality. It should also be noted that there may be cultural differences, particularly between countries, and so these results should not be thought to be globally comprehensive. Likewise, the relationship with other pets could be explored, since the activities reported are likely to differ from one species to another. Even though our framework has a good representability of dog owners (different ages, genders, dog-related expertise, dog sizes, dog ages and dog ownership duration) and saturation of themes has been reached in our thematic analysis, the framework cannot encompass all possible activities existent in all dog-owner dyads. More activities are expected to be found in studies targeting specific types of dog owners, i.e. those who have a particular goal associated with their relationship, such as a form of work or a specific hobby or interest. Any new activities might be rare at a general population level and/or not likely to change the well-being of dog owners at a population level. For example, even though some owners might be happy while dressing their dogs with different clothes and that might be a very important activity in their lives, this activity is not likely to have a strong effect on the well-being at a population level but could still be added into our framework to make it even more complete.

The creation of a definition for dog human related activities and the framework of activities and their well-being outcomes provided in this qualitative study provides a more robust basis for future research in the field examining the mental health of dog owners (or even in the field of dog-assisted interventions), which should help to resolve current inconsistencies. This new, more specific approach opens the door to the investigation of changes in human well-being which acknowledges the individuality of each dog-owner dyad. This framework also provides the foundation for the development of more robust study designs and treatment plans for patients with mental health issues, which might be helped by dog-assisted interventions or dog owners simply hoping to improve their mental health.

## Methods

### Participants

Initially, convenience and voluntary samplings were used to recruit self-identified dog owners via the University of Lincoln’s Petscando database (volunteer owners), social media, in person leaflet distribution and leaflets placed at pet shops, food markets and buildings of the university. 91 dog owners filled a recruitment form to volunteer to the study. Some of them were selected through purpose sampling to generate greater sample diversity with regards to owners’ age, gender, dog-related expertise or not (e.g. dog trainer, veterinarian of small animals, volunteer in dog-assisted interventions), size of their dogs, age of their dogs and duration of the dog ownership. Diagnosable mental health problems were not directly investigated in the study (e.g. as a selection criterium), owners with and without diagnosable mental health conditions were welcome to participate and, during the focus group, they were free to disclose as much or as little of their mental health status as they wanted. The population was thus mental health stigma-free, and accordingly honest and unbiased by such issues.

Based on the 91 registrations, a total of 45 participants were selected. Ten of them were not included as they either did not attend or arrived too late for the focus group meeting. The 35 dog owners who comprised the final sample, were aged from 18–24 years to 65–74 years old (median 25–34 years), 26 were female (74.3%), 15 owners had some dog-related expertise (42.9%), eight owners had small dogs (23%), 14 medium dogs (40%), 13 large dogs (37%), their dogs’ age ranged from a few months old to up to more than 15 years old (median 1–3 years old) and dog ownership lasted from a few months to more than 15 years (median 1–3 years). Finally, allocation to one focus group or another was performed according to participants’ time availability.

### Pilot study and focus group sessions

Focus groups consist of an organised discussion with a selected group of people to gather information on a topic^55^, and are recommended for exploratory research^56^, such as to the development of theoretical frameworks^57^, justifying their use in this work. This study was approved by the ethical review committee at the University of Lincoln (reference 2019-Jul-0503), and all methods were carried out in accordance with the university Research Ethics Policy and with the BPS Code of Ethics and Conduct. Written informed consent was obtained from all participants. A pilot session, with four volunteers, was conducted to assess the quality of the moderator guide and time management before the main focus groups. All focus groups took place at the University of Lincoln, UK and were focused on dog human related activities and their impact on well-being. They were moderated by the first author, who had been trained in focus group moderation and supported by an assistant. Two audio devices were used simultaneously for recording: Homder Digital Audio Recorder and iPhone 8 (Apple). In line with Guest et al.^58^, which reports that 90% of themes in focus groups are usually discovered within three to six sessions, a total of six sessions (besides the pilot) were performed in this study, and saturation of themes was achieved. Each of the 35 participants could take part in one session only. Group sizes ranged from four to eight dog owners, and session duration varied from 80–103 min (mean 91 min).

Based on a pre-defined semi-structured moderator guide, dog owners were first introduced to the concepts of dog human related activities, hedonic well-being, life satisfaction and eudaimonic well-being in a similar way they have been defined in the Introduction of this paper but with simpler words (e.g. instead of using the word affect in hedonic well-being, the moderator referred to it as emotions and moods) and through a PowerPoint presentation aimed to facilitate the comprehension of the concepts. Examples of dog human related activities during this explanation were minimal so as not to influence owners’ replies in the next step.

Second, owners were asked about the four most important activities for their own subjective well-being (positive and negative affect plus life satisfaction), and the four most crucial activities linked to their eudaimonic well-being. In order to prevent inter-participant bias and to generate a greater diversity of activities, dog owners were initially instructed to individually write these activities on a piece of paper provided. Participants were free to write more than one well-being outcome for each activity and it was made clear that they could also write less than four dog human related activities for each aspect of well-being if they wished. When asked about activities important to their life satisfaction and hedonic well-being, life satisfaction itself or any aspect of affect (e.g. happiness, sadness, calm) was a potential outcome. No predetermined fixed-options of affect was used to help participants characterise freely, with any word, the nature of the affect they experience from each activity reported, as recommended by Scherer^59^. In contrast, when participants wrote the activities essential for their eudaimonic well-being, they were asked to use the theory of the six elements of this well-being (e.g. autonomy, self-acceptance) as a guide for their reply, as these elements are part of the core concept of eudaimonic well-being.

Third, once all owners had finished writing, they shared out loud their list of dog human related activities and provided some background to justify their connection with the well-being outcome mentioned. At this point, dog owners had the opportunity to interact with each other, for example, discussing and elaborating on each other’s points. Several new dog human related activities emerged from that discussion and participants had the opportunity to agree or disagree with each other’s perceptions, which was important to evidence both positive and negative well-being outcomes of the same activity. In that discussion, probes and prompts were used by the moderator to clarify and gather further information from participants.

### Transcription and data analysis

Word-for-word transcription of the audio records and data analysis were performed on Microsoft Word and NVivo 11, respectively. Thematic analysis of the transcripts was conducted in three steps. First, dog owner related activities and elements of hedonic well-being, life satisfaction and eudaimonic well-being were coded. Second, activity codes were grouped according to their similarity in order to generate themes, e.g. ‘hiking’ and ‘running’ with the dog were grouped within the theme ‘exercise’. Affect elements were grouped based on the dimensional models presented by Russel^60^, Scherer^59^ and Yik et al.^61^, and eudaimonic elements were grouped according to Ryff’s^36^ classification. For example, the feelings ‘calm’ and ‘relaxed’ were grouped together as a hedonic aspect of positive valence-low arousal. At this stage, the final codes and themes were decided following discussion and consensus reached among the authors. Third, activities and themes were linked with their well-being outcomes through matrix coding (cross-tabulation of the frequency of references to each component). For example, the number of times the theme ‘tactile interactions’ was mentioned as beneficial to self-esteem (eudaimonic well-being) was calculated, as well as all other themes in relation to this well-being outcome. In order to have a better view of this cross-tabulation, a heat map was created (Fig. 2).

## Supplementary information

Supplementary information

## Data Availability

Data used for analysis are included in the supplementary information.
