# Supplementary material for: A framework for understanding how activities associated with dog ownership relate to human well-being
Source: Sci Rep. 2020 Jul 9;10:11363. doi: 10.1038/s41598-020-68446-9 (PMC7347561; doi:10.1038/s41598-020-68446-9)
Supplement: Supplementary file 1 — Supplementary information [file 41598_2020_68446_MOESM1_ESM.pdf]

A framework for understanding how activities associated with dog ownership relate to human well-being

Barcelos, Ana Maria[1]; Kargas, Niko[2]; Maltby, John[3]; Hall, Sophie[3]; Mills, Daniel S.[1]

[1]School of Life Sciences, University of Lincoln, Lincoln, United Kingdom
[2]School of Psychology, University of Lincoln, Lincoln, United Kingdom
[3]Department of Neuroscience, Psychology and Behaviour, University of Leicester, Leicester, United Kingdom

	Hedonia						Eudaimonia											Life sat.
	Increase in				Decrease in		Increase in						Decrease in					Increase in
	NvHa	NvLa	PvHa	PvLa	NvHa	NvLa	Aut	Env	Per	Pur	Pos	Sel	Aut	Env	Pur	Pos	Sel	LS
1. Aging and end of life of dog	4	6	0	1	1	0	0	0	0	0	0	0	0	1	0	0	0	0
Euthanasia, end of life - dog	3	4	0	1	0	0	0	0	0	0	0	0	0	0	0	0	0	0
Old dog - having it	1	2	0	0	1	0	0	0	0	0	0	0	0	1	0	0	0	0
2. Exercise with dog	2	0	32	23	9	5	7	7	3	10	24	2	0	0	0	0	0	3
Cycling	0	0	1	1	0	0	0	0	0	0	0	0	0	0	0	0	0	1
Hiking	0	0	1	1	1	0	1	0	0	0	1	0	0	0	0	0	0	0
Running	0	0	3	2	0	0	1	1	0	1	0	0	0	0	0	0	0	1
Sledging	0	0	1	1	0	0	0	0	0	0	0	0	0	0	0	0	0	0
Swimming	0	0	3	2	0	0	0	0	0	0	1	0	0	0	0	0	0	0
Walking	2	0	23	16	8	5	5	6	3	9	22	2	0	0	0	0	0	1
3. Failing to meet dog's needs, expectations	5	8	0	0	0	0	0	0	0	0	0	0	4	0	1	1	0	0
Leave dog alone, behind	**5**	7	0	0	0	0	0	0	0	0	0	0	4	0	1	1	0	0
Not fulfill dogs needs, e.g. walking	0	1	0	0	0	0	0	0	0	0	0	0	0	0	0	0	0	0
4. Grooming (theme)	3	0	7	4	0	0	0	0	0	1	0	1	0	0	0	0	0	0
Bath the dog	1	0	0	1	0	0	0	0	0	0	0	0	0	0	0	0	0	0
Clean dog's teeth	0	0	2	1	0	0	0	0	0	0	0	1	0	0	0	0	0	0
Grooming - non-specific	2	0	3	2	0	0	0	0	0	1	0	0	0	0	0	0	0	0
The result of grooming, e.g. dog is clean	0	0	2	0	0	0	0	0	0	0	0	0	0	0	0	0	0	0
5. Non-specific ownership routines	7	0	2	7	5	1	9	13	19	27	14	12	0	0	1	0	1	2
Having the dog, being a dog owner	1	0	2	6	2	0	4	6	13	11	14	7	0	0	1	0	0	2
Look after the dog, routine	3	0	0	1	3	1	5	7	6	16	0	5	0	0	0	0	0	0
Look after a puppy	3	0	0	0	0	0	0	0	0	0	0	0	0	0	0	0	1	0
6. Picture-related	0	0	4	1	0	0	0	0	0	0	0	0	0	0	0	0	0	0
Posting pictures, videos of dog on social media	0	0	3	0	0	0	0	0	0	0	0	0	0	0	0	0	0	0
Take photos of dog	0	0	1	1	0	0	0	0	0	0	0	0	0	0	0	0	0	0
7. Playing with dog	0	0	18	3	1	2	0	0	1	1	1	1	0	0	0	0	0	0
8. Providing for the dog	2	0	11	6	0	0	1	5	0	4	0	0	0	0	0	0	0	0
Buy snacks, treats for dog	0	0	2	1	0	0	0	0	0	0	0	0	0	0	0	0	0	0
Buy toys, objects to dog	0	0	2	1	0	0	0	0	0	0	0	0	0	0	0	0	0	0
Cook for dog	0	0	1	1	0	0	0	0	0	0	0	0	0	0	0	0	0	0
Feeding the dog	0	0	6	3	0	0	1	5	0	4	0	0	0	0	0	0	0	0
Take dog to the vet	2	0	0	0	0	0	0	0	0	0	0	0	0	0	0	0	0	0
9. Shared activity in the house	0	0	18	21	10	6	3	1	1	4	3	8	0	0	0	0	0	0
Being followed by the dog	0	0	0	1	0	0	0	0	0	0	0	0	0	0	0	0	0	0
Being greeted by dog	0	0	15	15	1	4	0	0	0	1	1	7	0	0	0	0	0	0
Dog waiting for owner to do something	0	0	1	1	0	0	0	0	0	2	1	1	0	0	0	0	0	0
Dog's presence in the house	0	0	2	4	9	2	3	1	1	1	1	0	0	0	0	0	0	0
10. Shared activity outside the house	1	0	14	3	13	7	8	1	3	2	19	3	0	0	0	0	0	1
Dog's company at work	0	0	1	1	1	0	1	1	2	0	1	1	0	0	0	0	0	0
Dog's presence while outside the house	0	0	11	1	10	6	4	0	1	1	17	2	0	0	0	0	0	0
Pet therapy with own dog	0	0	0	0	0	0	0	0	0	0	0	0	0	0	0	0	0	1
Trip with dog	1	0	2	1	2	1	3	0	0	1	1	0	0	0	0	0	0	0
11. Social interactions	16	0	14	9	5	5	0	0	2	0	31	2	0	0	0	7	0	0
Contact with other people, dog while out with your dog	10	0	9	2	4	4	0	0	1	0	12	2	0	0	0	3	0	0
Meeting with other dog lovers with or without dogs	0	0	4	2	1	1	0	0	0	0	11	0	0	0	0	0	0	0
Talk about dog	1	0	1	5	0	0	0	0	0	0	7	0	0	0	0	0	0	0
Talk to dog	0	0	0	0	0	0	0	0	1	0	0	0	0	0	0	0	0	0
Unappropriate interaction with your dog - from other person or dog	**5**	0	0	0	0	0	0	0	0	0	1	0	0	0	0	4	0	0
12. Tactile interactions (theme)	1	0	20	44	10	11	0	2	1	4	0	7	0	0	0	0	0	0
Being licked, kissed by dog	0	0	3	5	0	1	0	0	0	0	0	2	0	0	0	0	0	0
Cuddle, snuggle with dog	1	0	9	19	4	6	0	1	1	2	0	5	0	0	0	0	0	0
Pet the dog	0	0	0	1	1	0	0	0	0	0	0	0	0	0	0	0	0	0
Sleep, lie on bed with dog	0	0	4	12	4	2	0	1	0	1	0	0	0	0	0	0	0	0
Tactile interactions - non-specific	0	0	4	7	1	2	0	0	0	1	0	0	0	0	0	0	0	0
13. Teaching or learning	7	0	24	6	4	0	0	0	14	3	1	6	0	0	0	0	1	0
Agility	0	0	2	1	0	0	0	0	0	0	0	0	0	0	0	0	0	0
Learn dog-related things	0	0	1	0	1	0	0	0	8	0	1	1	0	0	0	0	0	0
Training, showing dog's skills	**7**	0	21	5	3	0	0	0	6	3	0	5	0	0	0	0	1	0
14. Unwanted behaviours	23	8	3	1	3	0	0	0	0	0	0	0	0	0	0	3	4	0
Aggression	2	1	0	0	1	0	0	0	0	0	0	0	0	0	0	1	1	0
Barking	**9**	0	0	0	1	0	0	0	0	0	0	0	0	0	0	1	2	0
Biting, trying to bite, lunging	3	0	0	0	1	0	0	0	0	0	0	0	0	0	0	1	1	0
Chewing, destroying objects	2	3	0	0	0	0	0	0	0	0	0	0	0	0	0	0	0	0
Farting	0	1	1	0	0	0	0	0	0	0	0	0	0	0	0	0	0	0
Growling	2	0	0	0	0	0	0	0	0	0	0	0	0	0	0	0	0	0
Poo related, e.g. roll on it, eat it, defecate in the house	3	1	0	0	0	0	0	0	0	0	0	0	0	0	0	0	0	0
Snoring - dog	1	2	0	0	0	0	0	0	0	0	0	0	0	0	0	0	0	0
Stealing, e.g. food, objects	1	0	2	1	0	0	0	0	0	0	0	0	0	0	0	0	0	0
15. Watch dog's behaviour	2	1	28	7	2	0	0	1	0	1	0	1	0	0	0	0	0	0
See dog in a situation (not described as positive or negative for the dog) that pleases the owner, e.g. funny noise while sleeping	0	0	11	1	1	0	0	0	0	0	0	1	0	0	0	0	0	0
See dog in a situation it dislikes	1	1	0	0	0	0	0	0	0	0	0	0	0	0	0	0	0	0
See dog in a situation it enjoys	1	0	17	6	1	0	0	1	0	1	0	0	0	0	0	0	0	0

Nv and **Pv** (negative and positive valence, respectively), **Ha** and **La** (high and low arousal, respectively), **Aut** (autonomy), **Env** (environmental mastery), **Per** (personal growth), **Pur** (purpose in life), **Pos** (positive relations), **Sel** (self-acceptance), **LF** (life satisfaction).
